# Exploring the coordination change of vanadium and structure transformation of metavanadate MgV_2_O_6_ under high pressure

**DOI:** 10.1038/srep38566

**Published:** 2016-12-07

**Authors:** Ruilian Tang, Yan Li, Shengyi Xie, Nana Li, Jiuhua Chen, Chunxiao Gao, Pinwen Zhu, Xin Wang

**Affiliations:** 1 State Key Laboratory of Superhard Materials, College of Physics, Jilin University, Changchun 130012, China; 2 Center for High Pressure Science and Technology Advanced Research, Changchun 130012, China; 3 Center for the Study of Matter at Extreme Condition, Department of Mechanical and Materials Engineering, Florida International University, Miami, FL 33199, USA

## Abstract

Raman spectroscopy, synchrotron angle-dispersive X-ray diffraction (ADXRD), first-principles calculations, and electrical resistivity measurements were carried out under high pressure to investigate the structural stability and electrical transport properties of metavanadate MgV_2_O_6_. The results have revealed the coordination change of vanadium ions (from 5+1 to 6) at around 4 GPa. In addition, a pressure-induced structure transformation from the *C2*/*m* phase to the *C2* phase in MgV_2_O_6_ was detected above 20 GPa, and both phases coexisted up to the highest pressure. This structural phase transition was induced by the enhanced distortions of MgO_6_ octahedra and VO_6_ octahedra under high pressure. Furthermore, the electrical resistivity decreased with pressure but exhibited different slope for these two phases, indicating that the pressure-induced structural phase transitions of MgV_2_O_6_ was also accompanied by the obvious changes in its electrical transport behavior.

Vanadium oxide-based compounds have received considerable attentions owing to their outstanding physical and chemical characteristics, which are originated from their diverse crystal structures, the various valance states of the vanadium ions in the compound, and also the different V-O coordination spheres^1^. Consequently, they could be used in extensive industrial applications, such as phosphors, gas sensors, catalysis, rechargeable batteries, and also in electrochemical devices^2^,^3^,^4^,^5^,^6^,^7^. A prominent example is that MnV_2_O_6_ with brannerite structure shows a high charge capacity when used as anode material for lithium ion rechargeable batteries^8^,^9^,^10^.

It is generally known that the interatomic distance of materials can be reduced under high pressure, resulting in significant changes in optical, electrical, and structural properties. In addition, the high pressure investigations on phase transition and phase stability of vanadium oxide-based compounds have led to high temperature and high pressure synthesis of novel phases^11^,^12^,^13^. Therefore, the high pressure behavior of vanadium oxide-based compounds has become a particularly attractive subject and a considerable amount of works have been focused on the structural phase transitions and physical properties of these compounds. Alkali metavanadates of *M*VO_3_ (*M* = Li, K, Rb, and Cs) have been reported to undergo structural phase transitions at moderate pressure of 5–20 GPa^14^,^15^,^16^,^17^, but only NaVO_3_ underwent pressure-induced amorphization at 6.1 GPa^18^,^19^,^20^. Previous studies of orthovanadates *A*VO_4_ including Raman scattering^21^, X-ray diffraction^22^,^23^, and combined methods^24^,^25^,^26^,^27^ have been carried out to understand the structural phase transitions induced by pressure. A “devil’s staircase”-type phase transition in the quarter-filled spin-ladder compound NaV_2_O_5_ has been discovered at low temperature and high pressure^28^. Interestingly, the pressure-induced superconductivity has been found in *β*-*X*^+^_0.33_V_2_O_5_ (*X*^+^ = Li, Na, and K) but absent in *β*-*X*^*2*+^_0.33_V_2_O_5_ (*X*^*2*+^ = Ca, Sr, and Pb), although a charge order transition existed in all these compounds except *β*-Pb_0.33_V_2_O_5_^29^,^30^,^31^,^32^.

In this context, divalent metal metavanadates *A*V_2_O_6_ (*A* = Mg, Ca, Sr, and Ba) are practical and attractive candidates for further investigations. Both SrV_2_O_6_ and BaV_2_O_6_ adopted an orthorhombic structure at ambient condition, and they have been reported to exhibit phase transition and amorphization under high pressure^33^. Known as an efficient catalyst, monoclinic MgV_2_O_6_ has been well investigated at ambient condition. In addition, different methods to synthesize MgV_2_O_6_ and its structure evolution under high temperature have been reported^12^,^34^,^35^,^36^,^37^. However, structural information and electrical transport properties of MgV_2_O_6_ under high pressure have not been studied.

At ambient condition, MgV_2_O_6_ adopts brannerite-like structure (space group *C2/m*). As shown in Fig. 1, the Mg^2+^ cations lie in octahedral sites, and the vanadium atoms occupy distorted VO_6_ octahedral sites, where it can be described as 5 + 1 coordination since the sixth oxygen atom is only weakly bonded at distances of 2.4–2.8 Å whereas the other oxygen atoms bonding below 2.4 Å^38^,^39^. The distorted VO_6_ octahedra are joined by sharing opposite corners forming chains along *b* axis of the unit cell. Pairs of octahedra in adjacent chains share edges on side of the chain.

In the present paper, we report a comprehensive investigation on the high pressure behavior of MgV_2_O_6_ at room temperature using Raman spectroscopy, ADXRD, first-principles calculations, and electrical resistivity measurements. A coordination change of vanadium ions from 5+1 to 6 and a structural phase transition (space group from *C2/m* to *C2*) have been found around 4 GPa and 20 GPa, respectively. The origins of this structural phase transition and subtle differences between these two structures have been analyzed in details. Significant changes in electrical resistivity have also been detected when the structural phase transitions occurred.

## Results and Discussion

Before compression, the crystal structure of the as-synthesized sample is characterized using XRD (see Supplementary Fig. S1). All diffraction peaks match well with the monoclinic structure MgV_2_O_6_ (space group *C2/m*, JCPDS PCPDFWIN, No. 34–13) with lattice parameters: *a* = 9.2957(1) Å, *b* = 3.5002(3) Å, *c* = 6.7411(2) Å, and *β* = 111.7(2)°.

### Raman Spectra at High Pressures

**Figure 2:**
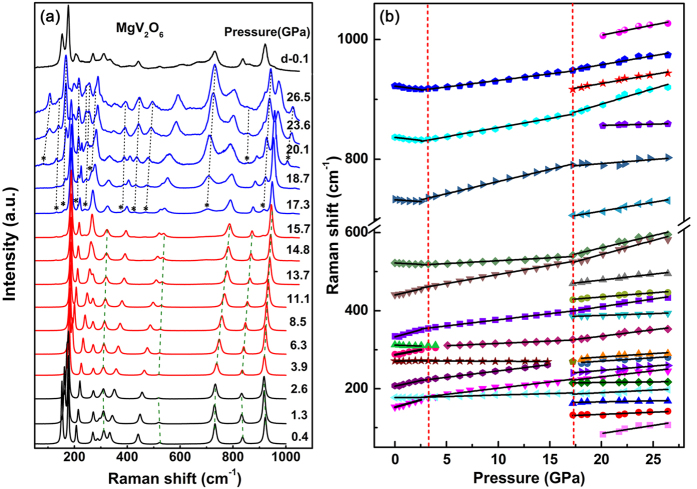
(**a**) Representative Raman spectra of MgV_2_O_6_ at high pressures. Asterisks represent the new Raman peaks. (**b**) Pressure dependence of Raman frequency shifts in MgV_2_O_6_. Vertical dotted lines indicate the phase transition pressures.

Representative Raman spectra of MgV_2_O_6_ at high pressures and the frequency shifts are shown in Fig. 2a and b, respectively. Factor group analysis gives the following irreducible representation of the optic modes Г_opt_ = 8A_g_(R) + 4B_g_(R) + 4A_u_(IR) + 8B_u_(IR) and the acoustic modes (A_u_ + 2B_u_) of MgV_2_O_6_^35^. Twelve phonon modes in total were discernible in the Raman spectrum at the ambient condition and the assignments of Raman bands, which are in good agreement with previous works^35^,^40^,^41^,^42^,^43^,^44^, are listed in Supplementary Table S1. In addition, the frequencies, pressure coefficients, and Grüneissen parameters of the different modes are summarized in Supplementary Table S2.

The strongest peak at 922 cm^−1^ is assigned to the stretching vibrations of V-O bonds. Peaks in the region of 500–750 cm^−1^ correspond to the stretching modes of V_3_O and (V_2_O_2_)_*n*_. The 288, 441 and 837 cm^−1^ peaks come from symmetric and antisymmetric stretching vibration of the V-O-V bonds along the VO_6_ octahedra chain, respectively. The band at 312 cm^−1^ is characteristic peak of V_3_O mode resulting from the edge-sharing VO_6_ octahedra between neighbouring chains. Three peaks located at 208, 271 and 334 cm^−1^ represent the lattice modes and MgO_6_ modes, respectively. Another two peaks located at 153 and 178 cm^−1^ corresponding to (V_2_O_2_)_*n*_ stretching modes generated by the edge-sharing between pairs of VO_6_ octahedra.

As shown in Fig. 2, most of the Raman modes continuously move to higher wavenumbers with pressure increased up to 26.5 GPa. On the contrary, five modes located at 312, 523, 733, 837, and 922 cm^−1^ firstly shift toward low frequency and then move to high frequency above 3.9 GPa. This change in the pressure dependence of Raman frequency without appearance of new Raman modes or disappearance of existing Raman modes indicates that MgV_2_O_6_ may undergo a subtle change in structure without altering symmetry at this pressure. With further increasing pressure, dramatic changes can be observed at 17.3 GPa and a set of new vibrational modes, which are marked by asterisks in Fig. 2a, appear at 132, 162, 214, 240, 384, 428, 469, 706, and 916 cm^−1^. Another three modes located at 82, 856 and 1006 cm^−1^ delay their appearance to a higher pressure (20.1 GPa). All the new Raman modes shift to the higher frequencies linearly accompanied by an increase in intensity upon further compression. These noticeable spectral changes under high pressure are attributed to structural phase transition of MgV_2_O_6_. Upon decompression, the spectroscopic changes are partially reversible and the high-pressure phase doesn’t fully revert to the original structure. In order to determine the structure of the high pressure phase of MgV_2_O_6_, *in- situ* ADXRD experiment was carried out and the structural evolution was also discussed in details in the subsequent section.

### ADXRD at High Pressures

**Figure 3:**
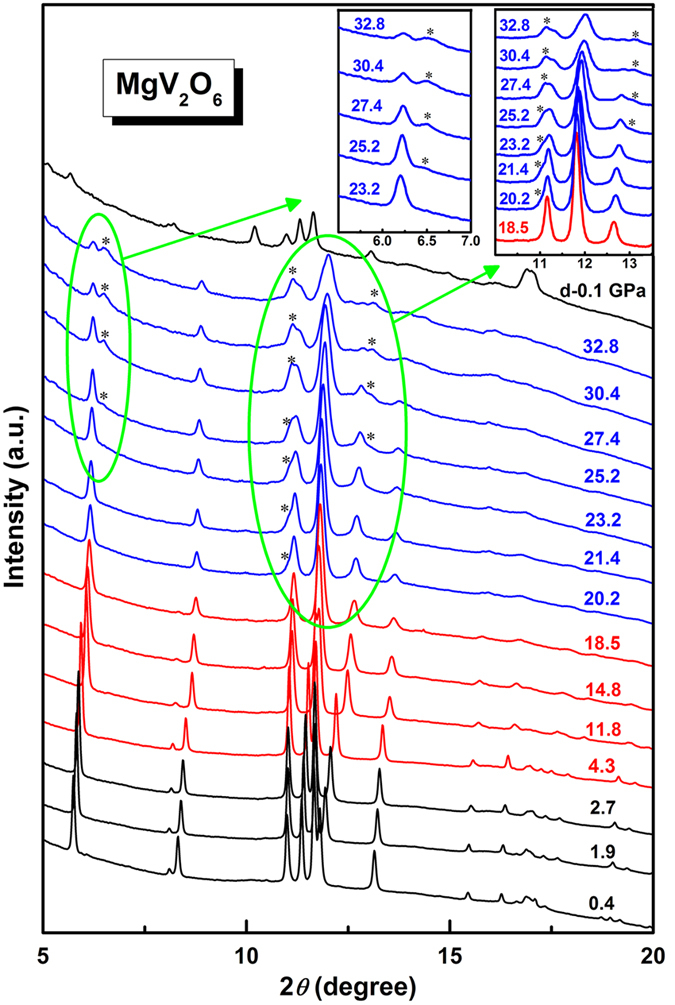
Selected high pressure ADXRD patterns of MgV_2_O_6_. Insert shows the appearance of the new peaks.

**Figure 4:**
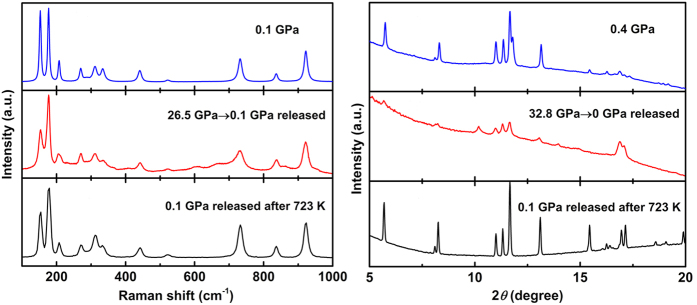
Raman spectra (left) and ADXRD patterns (right) of the sample at indicated conditions.

The ADXRD experiments were carried out up to 32.8 GPa and some selected patterns were shown in Fig. 3. All the reflections can be indexed by monoclinic structure MgV_2_O_6_ with space group *C2/m* and no new feature is observed below 18.5 GPa. In the pattern at 20.2 GPa, a new peak begin to emerge as marked by a star. This obvious change in the diffraction pattern corresponds to the onset of a structural phase transition which is also suggested by the Raman measurements. The observed small pressure difference may be related to the fact that Raman spectroscopy is more sensitive to local structural change than ADXRD^45^,^46^. Upon further compression, another two new diffraction peaks appear at 25.2 GPa. These three new peaks become gradually stronger with pressure although the peaks belonging to the original structure are still dominant even at the highest pressure reached. This suggests that there is a wide pressure range where the low- and the high-pressure phases coexist and the completion of this structural phase transition may require much larger pressure overstep. When the pressure released, the diffraction pattern doesn’t completely recover to the low pressure phase. It had been reported that some vanadates (NaVO_3_^19^, LiVO_3_^14^, SrV_2_O_6_, and BaV_2_O_6_^33^) can revert to their original phases from the high-pressure amorphous phases under high temperature. Therefore, in order to examine the reconstructing process of the structure, the pressure-quenched sample was heated in a muffle furnace at 723 K for 4 hours. Both Raman and XRD patterns indicate a recovery to the original phase after the heating, as shown in Fig. 4.

**Figure 5:**
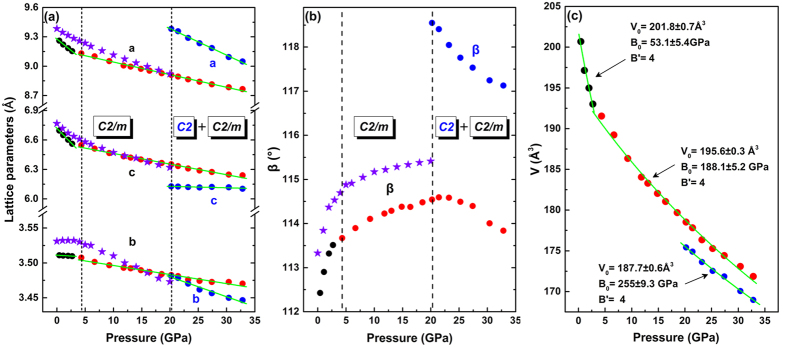
Pressure dependence of the lattice parameters (**a,b**) and volume (**c**) of MgV_2_O_6_. The circles represent experimental data and the stars represent theoretical calculation result.

Rietveld structural refinements were employed for accurate phase analysis using GSAS software and the high-pressure phase can be validated as another monoclinic structure with space group *C2*, as shown in Supplementary Fig. S2. In addition, the information of the refined crystal structure was presented in Supplementary Table S3. The derived lattice parameters and volume as a function of pressure are plotted in Fig. 5. Both unit cell dimension and volume decrease with pressure except the *β* angle which increases with pressure below 20.2 GPa. An obvious slope change in all the plots can be recognized at 4.3 GPa, indicating the occurrence of a subtle change in the structure of MgV_2_O_6_ which is also evidenced by Raman measurement.

The analyses of the V-O bond lengths as obtained from the structural refinement revealed that the longest V-O2(ii) bond length (2.6722 Å) drops dramatically under compression (down to 2.3733 Å at 4.3 GPa, as shown in Supplementary Fig. S3). This result is also reproduced by our calculations. At ambient pressure, six oxygen atoms are located around the vanadium atom and five of them have similar bond length (from 1.70 to 2.14 Å) and one has a noticeably longer bond length (2.61 Å). The longest V-O2(ii) bond length decreases rapidly under pressure (down to 2.4 Å at 4 GPa, as shown in Supplementary Fig. S3). Therefore, the abnormal change in compressibility and Raman vibration of MgV_2_O_6_ may be attributed to the coordination number of vanadium ions changes from 5 + 1 to 6 at about 4 GPa (forming a more rigid network). This phenomenon is different from the increase in coordination number of vanadium, which is caused by the conversion from tetrahedral VO_4_ to octahedral VO_6_ as occurred in InVO_4_ under pressure^47^. It is well known that the different catalytic properties of bulk and supported vanadate catalysts can usually be related to modifications in the coordination of the vanadium ions^48^. Therefore, the changes of V ions’ coordination number from 5 + 1 to 6 in MgV_2_O_6_ may lead to modify its redox properties.

**Figure 6:**
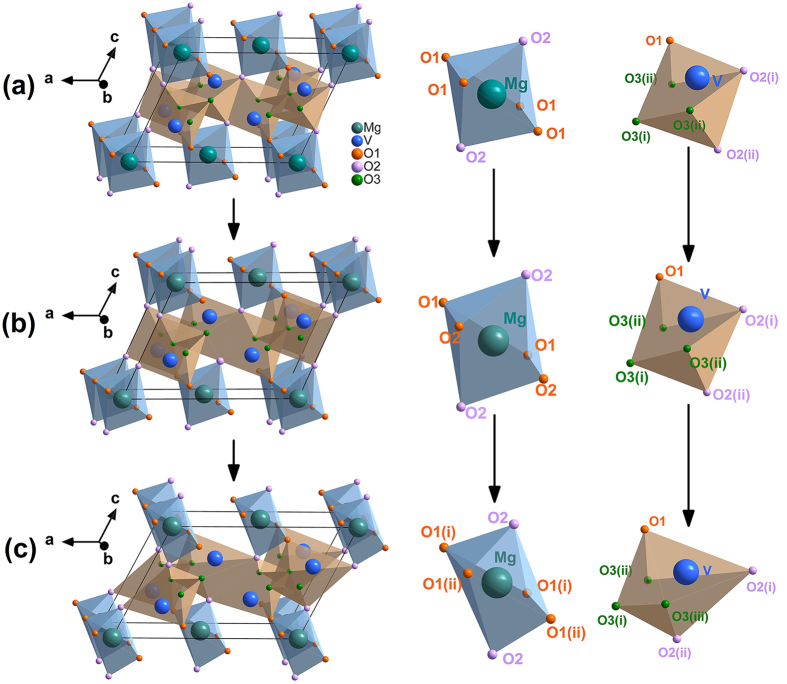
Structure of the *C2/m* phase at (**a**) 1.9 GPa, (**b**) 4.3 GPa, and the *C/2* phase at (**c**) 27.4 GPa, obtained from the Rietveld refinements of the XRD patterns.

The refined crystal structures of MgV_2_O_6_ in *C2/m* phase at 1.9 GPa, 4.3 GPa, and *C2* phase at 27.4 GPa based on the diffraction data are illustrated in Fig. 6. The derived Mg-O and V-O bond lengths of MgO_6_ octahedra and VO_6_ octahedra in different phases are listed in Table 1. It is worth noticing that the four identical Mg-O1 bonds in the MgO_6_ octahedra of the *C2/m* phase split into two groups in the *C2* phase, and so are the two V-O3 bonds in the VO_6_ octahedra. Figure 6 shows a pressure enhanced distortion of the MgO_6_ octahedra and VO_6_ octahedra in the *C2/m* phase which eventually leads to the structural phase transition from *C2/m* to *C2* phase. The *C2* phase described here is different from the recently reported new structure of MgV_2_O_6_ with space group *Pbcn* obtained at high temperature and high pressure^49^. The transition from *C2* to *Pbcn* phase may happen if temperature is elevated under high pressure.

The obtained pressure-volume data of MgV_2_O_6_ was fitted to a second order Birch–Murnaghan equation of state^50^ (fixed *B*′ = 4):


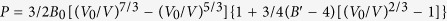


where *B*_*0*_ is the bulk modulus and *B*′ is its pressure derivative. The bulk modulus is determined to be *B*_*0*_ = 53.1 ± 5.4 GPa for the MgV_2_O_6_-*C2/m* phase (below 4 GPa), *B*_*0*_ = 188.1 ± 5.2 GPa for the MgV_2_O_6_-*C2/m* phase (above 4 GPa), and *B*_*0*_ = 255.0 ± 9.3 GPa for the MgV_2_O_6_-*C2* phase.

### Resistivity at High Pressures

**Figure 7:**
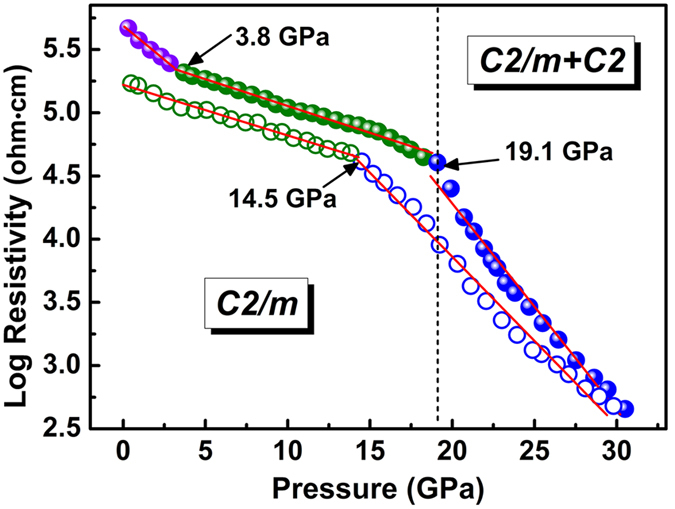
Resistivity as a function of pressure for MgV_2_O_6_ at room temperature.

As an important technical parameter, electrical resistivity, which can influences the application of the materials, is closely related to crystal structure. In addition, electrical resistivity is a more sensitive symbol for the electronic structural phase transition^51^,^52^. Therefore, we conducted electrical resistivity measurements at room temperature up to 30.5 GPa and the result is shown in Fig. 7. At ambient condition, the electrical resistivity of MgV_2_O_6_ is 4.66 × 10^5^ Ω·cm. Upon compression, the electrical resistivity decreases monotonously with pressure. An obvious kink point at 3.8 GPa is observed in the resistivity-pressure curve, which is caused by the coordination change of vanadium ions (from 5 + 1 to 6). Under further compression, another more significant kink occurs at 19.1 GPa. The sample resistivity quickly drops two orders of magnitude in the pressure range from 19.1 GPa to 30.5 GPa, indicating that the electrical resistivity of the *C2* high pressure phase is more sensitive to pressure. The qualitatively different behaviors of resistivity under compression are consistent with the stability of the two phases of MgV_2_O_6_ determined from XRD and Raman spectroscopy and the two pressure regions determined from the band-gap behavior (see Supplementary Fig. S4). The *C2/m* phase is an indirect band-gap semiconductor (E_g_ = 2.62 eV) which results a high resistivity at ambient condition. At 20 GPa a structural phase transition from *C2/m* to *C2* phase occurred, which it was associated with a band-gap collapse (E_g_ = 1.16 eV for *C2* phase). In both *C2/m* and *C2* phase, the band-gap always decreases under compression, resulting in the decline of the resistivity. This phenomenon is familiar with previous study in PbCrO_4_ under compression^53^. Upon decompression, the resistivity shows a reversible behavior across the *C2/m-C2* phase boundary with about 5 GPa hysteresis. In addition, both spectral (16:3:1 methanol/ethanol/water mixture) and electrical (without any pressure medium) results gave similar phase transition pressures, indicating that the deviatoric stresses has no effect on the structural phase transition in MgV_2_O_6_. Unlike the situation occurred in BaWO_4_ that the deviatoric stresses play an important role in its structural phase transition behavior^54^.

The integrated study using Raman spectroscopy, ADXRD, first-principles calculations, and electrical resistivity measurement indicate that MgV_2_O_6_ crystal structure experiences an effective coordination number change for vanadium cations from 5 + 1 to 6 at about 4 GPa and a structural phase transition from the *C2*/*m* phase to the *C2* phase at about 20 GPa upon compression. Pressure enhances the distortions of MgO_6_ octahedra and VO_6_ octahedra in the *C2/m* phase and ultimately results in the occurrence of the structural phase transition to *C2* phase. After the 5 + 1 to 6 coordination number change, the electrical resistivity of MgV_2_O_6_ becomes slightly less sensitive to pressure whereas the *C2* phase shows remarkable pressure induced decrease in its electrical resistivity.

## Methods

### Materials synthesis

The sample of MgV_2_O_6_ was prepared by sol-gel method. Highly pure V_2_O_5_ (99.99%), analytical grade Mg(NO_3_)_2_·6H_2_O, and oxalic acid (H_2_C_2_O_4_) were used as starting materials. A small pellet of the precursor was slowly heated in a muffle furnace and then cured at 873 K for 12 hours.

### High pressure Raman spectroscopy and high pressure angle-dispersive X-ray diffraction

A diamond anvil cell was used for applying high pressures to the sample. T301 stainless steel foil was used as a gasket. A methanol/ethanol/water mixture (16:3:1) was used as a pressure medium in both Raman spectra and *in-situ* ADXRD measurements and no pressure medium was used for electrical resistance measurement. The pressure in the sample chamber was measured by ruby luminescence technique^55^. High-pressure Raman spectra of MgV_2_O_6_ were carried out using a Jobin Yvon T64000 Raman microscope with a 633 nm He-Ne laser beam. High pressure ADXRD patterns were collected using MAR345 CCD detector at the 4W2 beam line of the Beijing Synchrotron Radiation Facility (BSRF) with wavelength of 0.6199 Å. Rietveld refinements were performed using the GSAS^56^ program to obtain the lattice parameters under pressure.

### High-pressure resistivity measurements

For the electrical resistivity measurements, a layer of mixture of cubic boron nitride (cBN) and epoxy were pressed onto the surface of the metal gasket for insulation. A hole of 120 μm in diameter was drilled at the center of the gasket for containing the sample using laser. Four hand-cut platinum foil strips of 10 μm thickness were directly attached to the sample as electrodes under a microscope (see Supplementary Fig. S5). The electrical insulation between the electrodes and the gasket was monitored during compression. The electrical resistivity of the sample was measured according Van der Pauw method^57^.

### First-principles calculations

Our first-principles calculations were performed with the projector augmented wave (PAW) method as implemented in the Vienna *ab initio* simulation package (VASP)^58^. The PAW pseudopotential was adopted with 2*p*^6^3*s*^2^, 3*p*^6^3*d*^3^4*s*^2^, and 2*s*^2^2*p*^4^ electrons as valence for Mg, V, and O atoms, respectively. The generalized gradient approximation add U (GGA + U) method with Perdew, Burke and Ernzerhof (PBE) type was used to describe the exchange and correlation interaction between the electrons. The Liechtenstein implementation with on-site Coulomb interaction U = 4.2 eV and on-site exchange interaction J = 0.8 eV was used to describe the localized *d* orbital of vanadium. The electronic wave functions were expanded in a plane-wave basis set with a cutoff energy of 700 eV. Monkhorst-Pack *k*-point meshes with a grid of 0.025 Å^−1^ for Brillouin zone sampling were chosen to achieve the total energy convergence of less than 1 meV/atom.

## Additional Information

**How to cite this article**: Tang, R. *et al*. Exploring the coordination change of vanadium and structure transformation of metavanadate MgV_2_O_6_ under high pressure. *Sci. Rep.* **6**, 38566; doi: 10.1038/srep38566 (2016).

**Publisher's note:** Springer Nature remains neutral with regard to jurisdictional claims in published maps and institutional affiliations.

## Supplementary Material

Supplementary Information

## Figures and Tables

**Figure 1:**
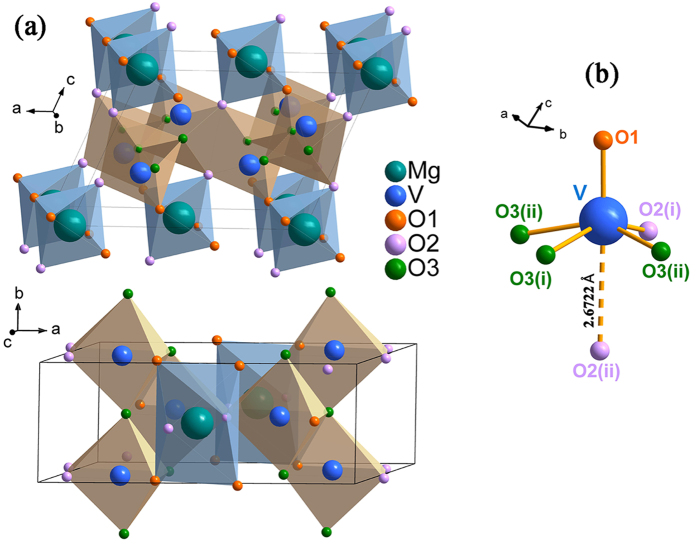
(**a**) Schematic view of the crystal structure and (**b**) V-O bonding diagram of MgV_2_O_6_ (space group *C2/m*).

**Table 1:** Mg-O and V-O bond lengths in MgO_6_ octahedra and VO_6_ octahedra for the *C2/m* phase at 1.9 GPa, 4.3 GPa, and *C2* phase at 27.4 GPa.

Pressure	MgV_2_O_6_	Mg-O distance (Å)		V-O distance (Å)		Figure
1.9 GPa	*C2/m*	**Mg-O1**	**2.2962**	V-O1	1.5192	6(a)
		**Mg-O1**	**2.2962**	V-O2(i)	1.5021	
		**Mg-O1**	**2.2962**	V-O2(ii)	2.4256	
		**Mg-O1**	**2.2962**	V-O3(i)	2.3551	
		Mg-O2	2.0031	**V-O3(ii)**	**1.9249**	
		Mg-O2	2.0031	**V-O3(ii)**	**1.9249**	
4.3 GPa	*C2/m*	**Mg-O1**	**2.1683**	V-O1	1.6873	6(b)
		**Mg-O1**	**2.1683**	V-O2(i)	1.6665	
		**Mg-O1**	**2.1683**	V-O2(ii)	2.3733	
		**Mg-O1**	**2.1683**	V-O3(i)	2.3126	
		Mg-O2	1.8748	**V-O3(ii)**	**1.9079**	
		Mg-O2	1.8748	**V-O3(ii)**	**1.9079**	
27.4 GPa	*C2*	**Mg-O1(i)**	**2.0790**	V-O1	1.6068	6(c)
		**Mg-O1(i)**	**2.0790**	V-O2(i)	1.5695	
		**Mg-O1(ii)**	**2.1849**	V-O2(ii)	2.2281	
		**Mg-O1(ii)**	**2.1849**	V-O3(i)	2.3725	
		Mg-O2	1.7279	**V-O3(ii)**	**1.8290**	
		Mg-O2	1.7279	**V-O3(iii)**	**2.1746**	
