# Supplementary material for: Exploring the coordination change of vanadium and structure transformation of metavanadate MgV2O6 under high pressure
Source: Sci Rep. 2016 Dec 7;6:38566. doi: 10.1038/srep38566 (PMC5141448; doi:10.1038/srep38566)

**Exploring the coordination change of vanadium and structure transformation of metavanadate MgV_2_O_6_ under high pressure**

Ruilian Tang^1,2^, Yan Li^1,^^*^, Shengyi Xie^2^, Nana Li^1,2^, Jiuhua Chen^2,3, *^, Chunxiao Gao^1^, Pinwen Zhu^1^, and Xin Wang^1,^^*^

^1^*State Key Laboratory of Superhard Materials, College of Physics, Jilin University, Changchun 130012, China*

^2^*Center for High Pressure Science and Technology Advanced Research, Jilin University, Changchun 130012, China*

^3^*Center for the Study of Matter at Extreme Condition, Department of Mechanical and Materials Engineering, Florida International University, Miami, FL 33199, USA*

^*^Corresponding author: xin_wang@jlu.edu.cn

liyan2012@jlu.edu.cn

chenjh@hpstar.ac.cn

Phone: +86-431-85168881.

Fax: +86-431-85168881.

**Supplementary Information**

**Figure S1**: The observed, calculated, and difference XRD patterns of MgV_2_O_6_ at ambient condition.


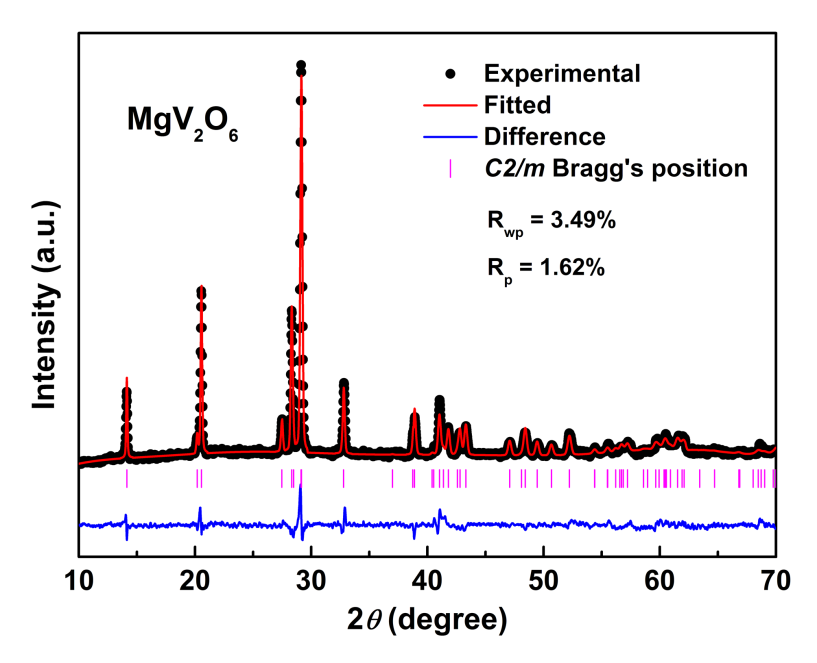


**Table S1.** Observed Raman frequencies and band assignments for MgV_2_O_6_ at ambient conditions.

| **Reference^35, 40-44^** | **This study** | **Assignment** |
| --- | --- | --- |
| 923 | 922 | *ν*(VO)*^str^* |
| 836 | 837 | *ν^as^*(VOV)*^str^* |
| 731 | 733 | ν(V_2_O_2_)*_n_*+ν(V_3_O)*^str^* |
| 523 | 523 |  |
| 440 | 441 | *ν^s^*(VOV)*^str^* |
| 332 | 334 | MgO_6_ modes |
| 309 | 312 | ν(V_3_O)*^str^* |
| 286 | 288 | *ν*(VOV)*^str^* |
| 268 | 271 | MgO_6_ modes |
| 204 | 208 | Lattice modes |
| 174 | 178 | ν(V_2_O_2_)*_n_* |
| 149 | 153 | ν(V_2_O_2_)*_n_* |
| *^str^* = stretch; *^as^* = antisymmetric stretch; *^s^* = symmetric stretch | | |

**Table S2.** The frequencies, pressure coefficients, and Grüneissen parameters of the Raman modes in MgV_2_O_6_. The Grüneissen parameter has been calculated using the bulk modulus as indicated in the text.

| ***C2/m* (blow 3.9 GPa)** | | |  | ***C2/m* (above 3.9 GPa)** | | |  | ***C2/m +C2*** | |
| --- | --- | --- | --- | --- | --- | --- | --- | --- | --- |
| ***ω*** | ***dω*/*d*P** | ***γ*** |  | ***ω*** | ***dω*/*d*P** | ***γ*** |  | ***ω*** | ***dω*/*d*P** |
| 922 | -2.27 | -0.13 |  | 918 | 2.18 | 0.45 |  | 1006 | 3.39 |
| 837 | -2.01 | -0.13 |  | 834 | 3.07 | 0.69 |  | 949 | 2.99 |
| 733 | -1.22 | -0.09 |  | 735 | 4.06 | 1.04 |  | 917 | 2.99 |
| 523 | -1.38 | -0.14 |  | 519 | 1.46 | 0.53 |  | 877 | 5.13 |
| 441 | 6.02 | 0.72 |  | 460 | 4.65 | 1.90 |  | 856 | 0.43 |
| 334 | 7.19 | 1.14 |  | 355 | 3.14 | 1.66 |  | 790 | 1.37 |
| 312 | -0.86 | -0.15 |  | 311 | 1.20 | 0.73 |  | 706 | 2.94 |
| 288 | 5.12 | 0.94 |  |  |  |  |  | 542 | 6.25 |
| 271 | 0.39 | 0.08 |  | 271 | 0.12 | 0.08 |  | 525 | 7.23 |
| 208 | 5.73 | 1.46 |  | 224 | 3.34 | 2.80 |  | 469 | 2.99 |
| 178 | 0.16 | 0.05 |  | 178 | 0.76 | 0.80 |  | 428 | 2.14 |
| 153 | 7.47 | 2.59 |  | 177 | 3.17 | 3.37 |  | 399 | 3.94 |
|  |  |  |  |  |  |  |  | 385 | 0.92 |
|  |  |  |  |  |  |  |  | 326 | 3.29 |
|  |  |  |  |  |  |  |  | 275 | 1.73 |
|  |  |  |  |  |  |  |  | 265 | 1.74 |
|  |  |  |  |  |  |  |  | 240 | 2.23 |
|  |  |  |  |  |  |  |  | 221 | 3.07 |
|  |  |  |  |  |  |  |  | 214 | 0.31 |
|  |  |  |  |  |  |  |  | 188 | 1.33 |
|  |  |  |  |  |  |  |  | 162 | 0.50 |
|  |  |  |  |  |  |  |  | 133 | 1.10 |
|  |  |  |  |  |  |  |  | 83 | 4.11 |

**Figure S2**: Rietveld full-proﬁle refinements of the diffraction patterns for MgV_2_O_6_ collected at (a) 1.9 GPa, (b) 4.3 GPa, and (c) 27.4 GPa.


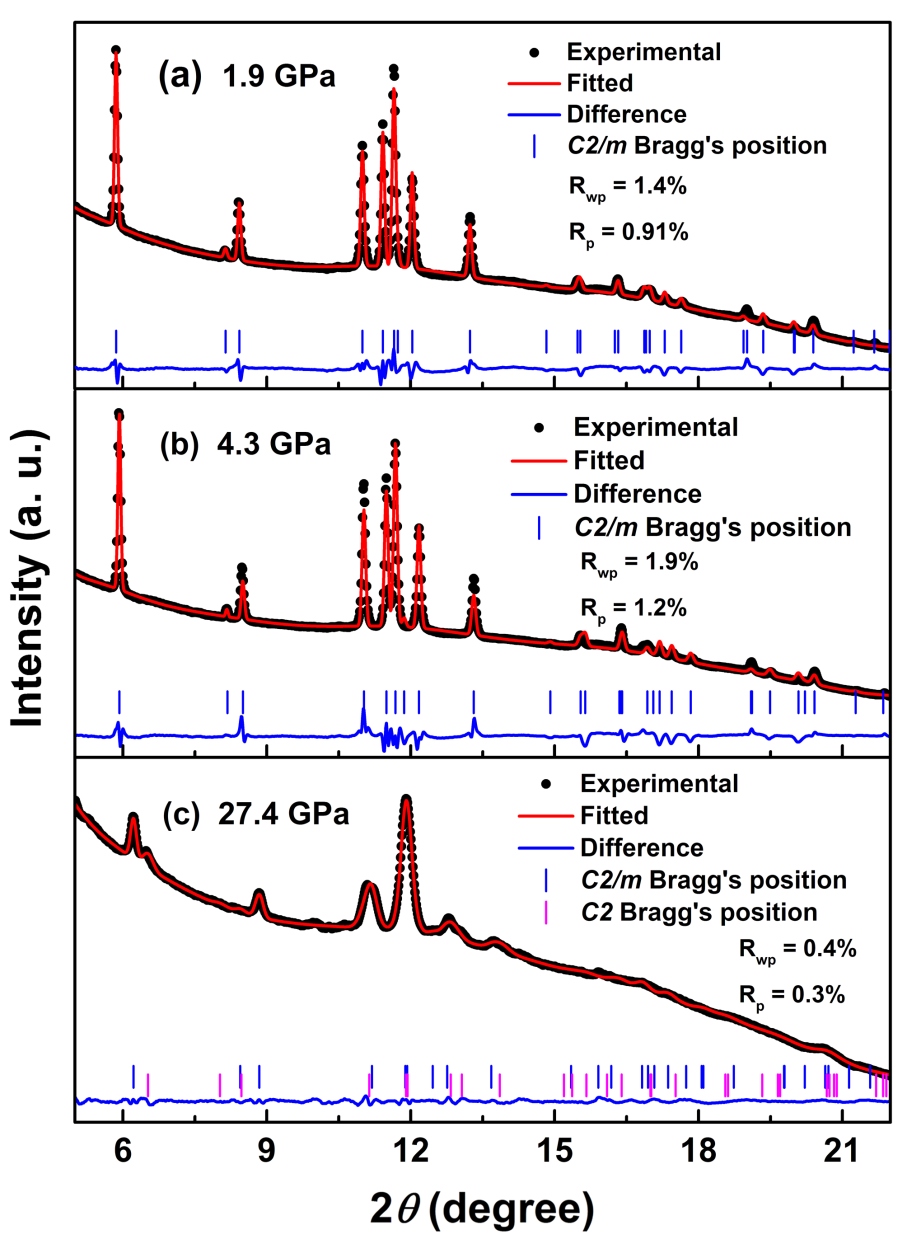


**Table S3**. Refined crystal structure of *C2/m* and *C2* phase of MgV_2_O_6_.

|  | ***C2/m*** | ***C2/m*** |  | ***C2*** |
| --- | --- | --- | --- | --- |
| Pressure | 1.9 GPa | 4.3 GPa |  | 27.4 GPa |
| *a* (Å) | 9.1838(3) | 9.1292 |  | 9.1855 |
| *b* (Å) | 3.5018(4) | 3.4975 |  | 3.4466 |
| *c* (Å) | 6.6028(1) | 6.5490 |  | 6.1219 |
| *β* (deg) | 113.3(1) | 113.6689 |  | 117.5340 |
| *V* (Å^3^) | 195.02(7) | 191.52 |  | 171.86 |
| Z | 2 | 2 |  | 2 |
| Mg (2a) | (0,0,0) | (0,0,0) | Mg (2a) | (0,0,0) |
| V (4i) | (0.3303, 0.5, 0.3302) | (0.3259, 0.5, 0.3345) | V (4c) | (0.6393, 0.4703, 0.3187) |
| O1 (4i) | (0.1735, 0.5, 0.1339) | (0.1517, 0.5, 0.1133) | O1 (4c) | (0.1541, 0.51911, 0.1100) |
| O2 (4i) | (0.4782, 0.5, 0.2901) | (0.4940, 0.5, 0.2828) | O2 (4c) | (0.4954, 0.4358, 0.2767) |
| O3 (4i) | (0.1930, 0.5, 0.5652) | (0.1930, 0.5, 0.5652) | O3 (4c) | (0.1803, 0.5053, 0.5315) |

**Figure S3**: Bond length of V-O2(ii) at different pressure: experiment and calculation data. Insert is the V-O bonding diagram at 0 GPa and 4.3 GPa.


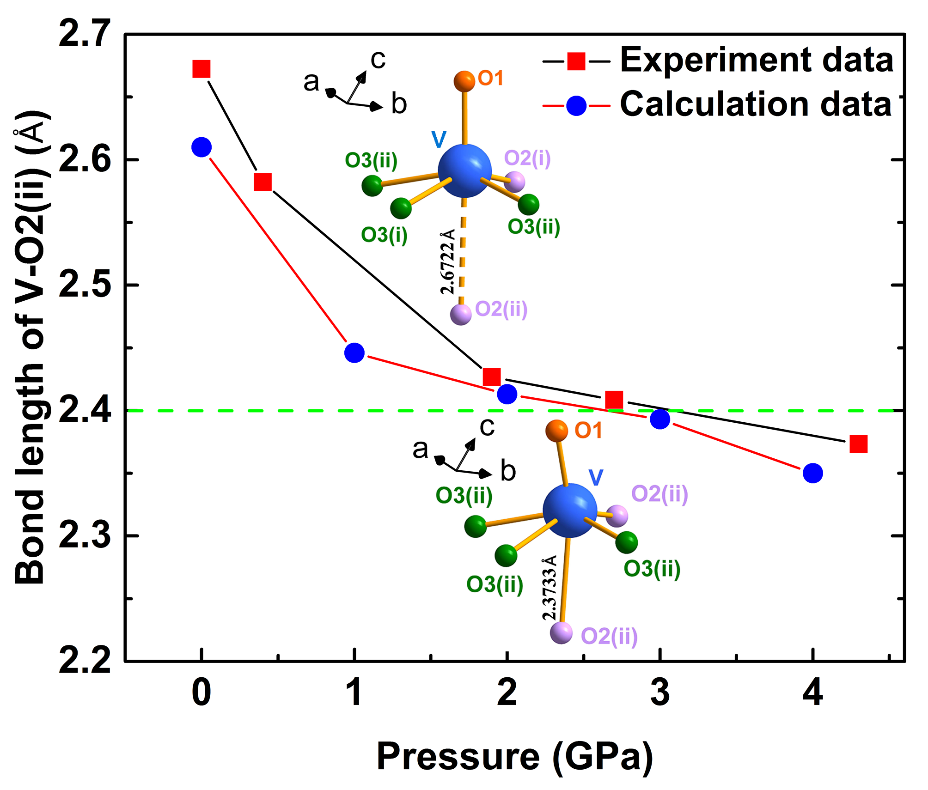


**Figure S4**: Calculated band structure of MgV_2_O_6_ at (a) ambient pressure for *C2/m* phase and (b) 30 GPa for *C2* phase. (c) Pressure dependence of band gap (E_g_).


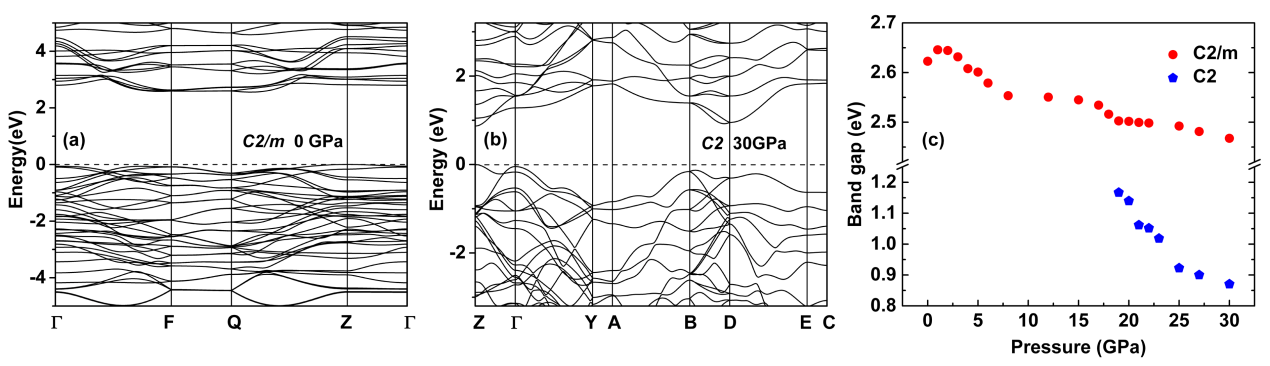


**Figure S5**: (a) Schematic drawing of DAC and the arrangement of sample and electrodes on the gasket. (b) Top view of the designed DAC. (c) A photograph of the sample and electrodes under pressure of 19.1 GPa.


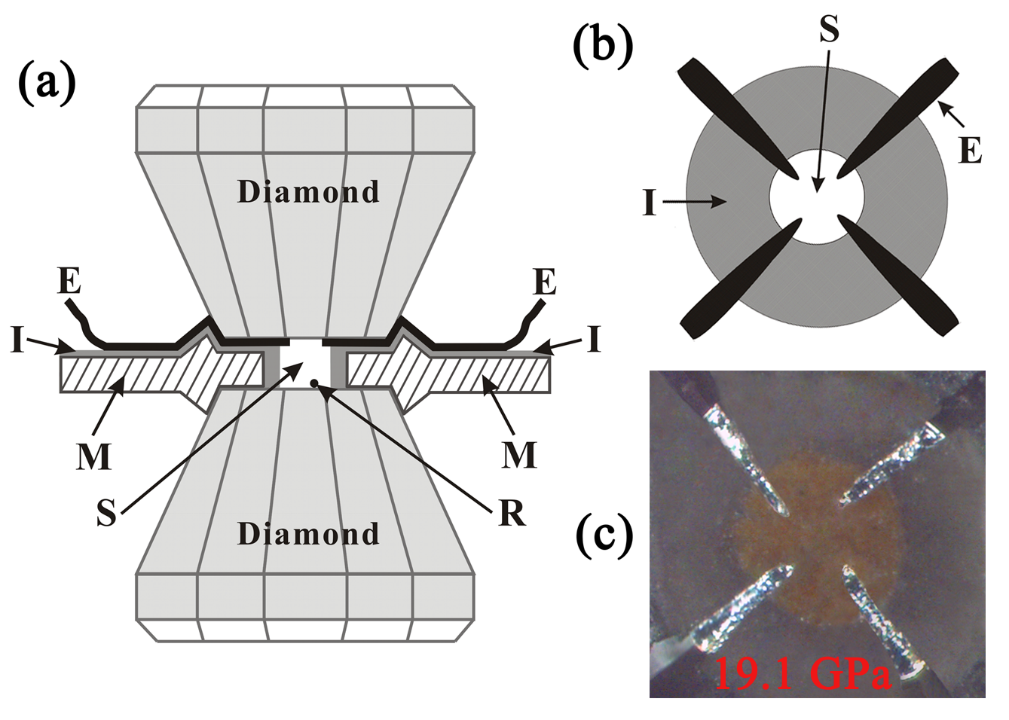

Supplement: Supplementary Information [file srep38566-s1.docx]
